# Explaining the process of formation of ageism among the iranian older adults

**DOI:** 10.1186/s40359-023-01153-y

**Published:** 2023-04-18

**Authors:** Ameneh Yaghoobzadeh, Parvaneh Asgari, Alireza Nikbakht Nasrabadi, Jila Mirlashari, Elham Navab

**Affiliations:** 1 School of Nursing and Midwifery, Arak University of Medical Sciences, Arak, Iran; 2 Department of Critical Care Nursing, School of Nursing and Midwifery, Tehran University of Medical Sciences, Tehran, Iran; 3 Department of Medical and Surgical Nursing, Tehran University of Medical Sciences, Tehran, Iran; 4 Department of OBGYN, Women’s Health Research Institute, University of British Columbia, Vancouver, Canada; 5 School of Nursing and Midwifery, Tehran University of Medical Sciences, Tehran, Iran; 6 Department of critical care and Geriatric nursing, School of Nursing and Midwifery, Tehran University of Medical Sciences, East Nosrat Street, Tohid Square, Tehran, 1419732171 Iran

## Abstract

**Background:**

Ageism is considered as one of the consequences of the industrialization of societies, which appears in various forms in different cultures. This study aimed to explain the process of formation of ageism among the older adults people.

**Methods:**

The research was conducted using grounded theory method. Data were collected from 28 participants using in-depth semi-structured interviews and field notes. Data were analyzed using open, axial, and selective coding.

**Results:**

Fear of loneliness and rejection striving to tackle ageism “was identified as the core category of the study. Concepts such as “family context” and “cultural context” were relevant. After identifying the strategies used by the older adults in response to the context (“maintaining integrity”, “socio-cultural care” and “proper health care”, “striving to tackle ageism”) was the most important process in ageism by the Iranian older adults.

**Conclusion:**

Findings of this study indicated that individual, family and social factors play an important role in the process of ageism among the older adults. These factors sometimes exacerbate or moderate the process of ageism. By recognizing these factors, various social institutions and organizations (including the health care system and the national media (radio and television)) can help the older adults achieve successful aging by emphasizing the issues related to the social aspect.

## Background

Aging is a period of life in which older people are exposed to potential threats such as chronic conditions, loneliness, isolation, lack of social support, and a decline in independence [1]The population of older people is rapidly growing worldwide. It is estimated that the population over the age of 60 will increase from 900 million in 2015 to 2 billion in 2050 [2]. The United Nations estimated that based on the assumption of moderate growth, by the 2040 and 2050 s, about 25% of the population of Iran will be over the age of 60 years [3]older adults. Increasing the proportion of older people to the general population will affect all aspects of social life from health to social security, environmental issues related to education, job opportunities, social and cultural activities, family life and legal domains [4].

Old age is one of the main stages of life and according to some, it is the end. Some people think that when they reach old age, they have nothing to do in the world anymore and they have to sit in a corner and wait for the end of their life. It is true that this stage of life coincides with the weakness of physical strength and the occurrence of some diseases, but it does not mean giving up effort, activity and hope. Therefore, entering old age is accompanied by the experience of a series of positive and negative events that a person has views about himself/herself as a result of the passage of time, or in some cases these views are instilled in him by the environment and people around him/her [5].

It seems, all of these cases are associated with the experience of ageism among the older adults. Ageism is a multidimensional concept that encompasses cognitive (stereotype), affective (prejudice) and behavioral (discrimination) components; positive and negative dimension; conscious and subconscious aspects and micro, meso and macro levels [6]. Ageism in different societies and cultures manifests itself in various forms which includes both positive and negative dimensions [7]. Kindness, wisdom, trust, feeling of freedom and happiness are defined as a positive attitude towards the older adults and perceptions such as presence of chronic disease, physical and mental disorders due to old age, depression and inadequacy in caring for grandchildren as negative attitudes [8].

Despite advances in medical sciences and improved quality of health care services, there are still barriers to providing high-quality services to the older adults. One of the barriers is the presence of negative stereotypes and attitudes towards aging that may reduce the efficiency and quality of these services [9]older adultsolder adults. In fact, community members believe that illness and disability are often associated with old age and the older adults do not need to be cared for [10, 11]. There has also been evidence of ageism in terms such as the boomer remover in relation to Covid-19. The boomer remover hashtag has been prevalent even on social media platforms, which has often been associated with patterns of devaluing the older adults (23). Ageism against the older adults will have many negative consequences. For example, the older adults people see themselves as a social burden and begin to experience a feeling of “waiting to die”. It is obvious that the existence of such negative stereotypes as well as the older adults person’s understanding, also affect his physical activities and social participation [12]. On the other hand, the onset of depressive symptoms, decreased self-efficacy, increased abuse, decreased self-esteem and decreased quality of life are other negative effects of ageism against this group of people [13–15]. Various studies have pointed out that family, social, sexual life and health care systems are the most common contexts in which discrimination against the older adults has been observed. Consequently this can reduce the chances of employment in the institution, less attention and respect in educational and medical centers [16]. In health care and nursing, ageism varies through a variety of mechanisms, from social stereotypes such as poor knowledge of care for the older adults to policies that reinforce inattention to this group of people [17]. The manifestation of ageism in nursing education, by prioritizing other specialties and skills, diverts students from their interest in caring for the older adults and deprives them of the possibility of promoting knowledge of the field and positive attitudes [18]. Existence of ageism in medical settings among nurses, especially in the field of care or having negative attitudes towards the older adults can provide grounds for abuse and neglect of the older adults [19]. Among different groups of populations, the older adults should be protected from abuse, and primary care nurses are central in detecting suspected cases of abuse and making appropriate interventions. In fact, nurses as a whole have a responsibility to address ageism and to influence the improvement of policies, rules and measures to ensure the safety and equality of older people. By adopting this multilevel approach, nurses will not only protect the older adults but also secure the future of themselves and others [20]. The experience of the Corona virus pandemic has raised new awareness of ageism around the world [21]. Nurses who provide long-term care to the older adults have certainly faced some of the most devastating effects of ageism. In fact, in the context of the corona-virus pandemic, the death of the older adults or the infection of these people due to their old age is considered normal, which is a clear example of ageism. Nurses, on the other hand, must be committed to eliminating ageism and replacing it with age awareness, increasing public perceptions of aging, and adhering to a set of principles and rules.

Iran is one of the ancient countries in the Middle East, representing more than 2500 years of civilization. Iranian culture emphasises altruism and strong family ties as two basic values. This makes Iranians feel more committed to relatives [22].

In the traditional cultures of Iran, the elderly are described as valuable people, and this valuation is due to their essential role in the transmission of knowledge, wisdom and experience [12].

Of course, the realistic approach regarding the current sociological changes in traditional cultures shows the difference in the attitude of honoring the elderly. In today’s lives, due to the industrialization of societies and the creation of social and economic changes, lifestyle, technology and difficult living conditions, the value of the elderly has decreased [23].

Considering the fact that in addition to Western cultures, in developing Asian cultures where the older adults were considered as respected and valued counselors, they also experienced some degree of discrimination [23]. It is important to explain and describe how this phenomenon occurs. Also, according to Butler’s definition of this phenomenon as being a process and the involvement of individual and social self and symbolic interactions in creating, intensifying, modifying or managing the phenomenon of ageism, having a mental nature and its impact on structures related to Community, explaining this phenomenon from the perspective of the older adults seems necessary. Therefore, this grounded theory study was conducted with the aim of explaining the formation process of ageism in the older adults and providing a suitable model for itolder adults.

## Methods

### Study design

The present study is a qualitative grounded theory research between 2020 and 2021. Considering the processing nature of ageism and the unique role of individuals and symbolic interactions in creating, intensifying or modifying it in the socio-cultural context of societies, grounded theory method was used to have a deep understanding of how ageism is formed and to determine the different dimensions of this phenomenon from the words of those who have experienced it.

Grounded theory scholars take a systematic approach to qualitative research, focus on theory development, engage in constant comparison and prioritize abductive reasoning by engaging in simultaneous data collection and analysis. Thus, grounded theory methods provide an opportunity to move beyond descriptions of qualitative data and extend to theorizing about the actions and processes in specific contexts, which aligns with the goals of our process-focused research question [24].

### Setting and participants

In the present study, 32 interviews with 28 participants (20 older adults, one adolescent, one young, one informal caregiver (older adults family members), three formal caregivers (one nursing expert, one geriatric nurse and one geriatrician), two Older adults policymakers who were able to express themselves well and were willing to participate in the research. The researcher tried to include people who had different experiences in the field of ageism in terms of age, sex, marriage, education, socio-economic status, employment status and place of residence in the study in order to obtain maximum diversity and theoretical saturation. The first three samples who were older adults were purposefully selected and the other participants were selected theoretically based on the findings of data analysis. Some participants were interviewed more than once.

### Data collection

After obtaining the necessary permits and informed consent, the time and place of the interview were determined in coordination with the participants. Data were collected through in-depth semi-structured interviews and field notes. In the present study, a set of questions related to the research topic were used to guide the interview and data collection. Accordingly, the interview began with general questions. For example, early interviews began with the questions: “What is your experience of old age?“, “How do you feel about your age?“, “How do people around you see you as an older adults person?“ During the interviews, other questions were asked as needed to clarify the theory being formed. Some of these questions include: “How have your relationships changed since then?“, “How do you connect with young people?“ “How do you see your role as an older adults person in society?“ Or “What changes have happened in your personal life as you get older?“ “What factors make you more/less social activity? Then, based on the answers and data presented by the participant, clarifying and in-depth interview questions such as “Please explain more in this regard. What does this mean? continued. Interviews were conducted according to the participant’s prior agreement and the time and place they were comfortable (. The duration of each interview was between 30 and 45 min. Also, in order to complete the data and examine the different dimensions of the phenomenon in natural conditions, field notes were used. The focus of the field notes was on how ageism was used against the older adultsolder adults, and in particular on the behaviors that could add to this discrimination. These notes focused on the physical space of the place where people live, their mentality and especially their interactions. Each interview taught the researcher points that could help gain a better understanding of the phenomenon being studied and to consider what should be included in the next interview. The researcher recorded all the interviews using an MP3 recorder and transcribed verbatim immediately after the interview. Constant comparison technique between data with the aim of extracting emerging categories directed the researcher to continue the theoretical sampling process until the saturation of data in each class was achieved.

### Data analysis

For data analysis, the paradigm model of Strauss and Corbin (2008) was used. Data analysis followed on constant comparative process with analysis, sampling and data collection occurring concurrently and informing each other. Open, axial and selective coding was used to analyzed the data. A process of microanalysis was first conducted by open coding of line-by-line audio transcripts. Initial codes were in-vivo, i.e.; in the words of the participants themselves. Once categories were identified to create categories by grouping similar concepts and exploring relationships between codes. Finally, we used selective coding to identify the core category and construct the conceptual model. In addition, constant comparative analysis was used to understand interactions among categories and variation in participant’s experience.

### Rigor

Multiple strategies were used to maintain rigor in data collection and analysis. Member checking was used throughout to determine whether the conceptual model accurately describe participants’ experiences (some participants were asked to review the results to ensure that the results matched their experience). Qualitative research team analyzed the data to reduce individual bias and improve credibility of the results. Also, to deepen the results of the research team, he tried to extract the common language of the study population by using multiple quotations in the results section and provide a clear picture of this process. Efforts were also made to extract the necessary questions to collect data from their statements by being sensitive to the reactions and statements of the participants and not to impose assumptions and ideas of researchers on the data. Additionally, we kept detailed methodological and theoretical memos to create an audit trial of all procedures including data analysis and sampling decisions to improve credibility and reproducibility of results.

### Ethical consideration

The present study was registered with the code of ethics (IR.TUMS.FNM.REC.1397.135) in the research ethics committee of Tehran University of Medical Sciences. The objectives and methods used in the study were fully explained to the participants. Participants were reassured that the content was kept confidential and anonymous. They were also told that participation in the study was optional and that it was possible to cancel the study at any time.

## Results

In the present study, there were 28 participants with the average age of 60.53 ± 19.48 of whom 11 were male and 17 were female. Given that the old age in developing countries is considered 60 years, the minimum age of older adults participants in this study was 60 years.

A central phenomenon in the process of ageism in the older adultsolder adults was “Fear of loneliness and rejection: striving to tackle ageism.“ Concepts such as “family context” and “cultural context” were relevant. Analysis of aging experiences showed that their main concern was confrontation with “fear of loneliness and rejection.“ In fact, entering the world of old age, the older adultsolder adults realize the difference in the type of behaviors and actions of people around them. Overcoming the physical and psychological problems associated with this period of life and differences in the type of social views of those around them cause them to consciously take steps to ensure their acceptance as much as they can.

### Discriminatory paradox

The “discriminatory paradox”, including “self-discrimination” and “other discrimination” was the first factor underlying ageism in the older adultsolder adults. Entering old age is accompanied by the experience of a series of positive and negative events in which one gain perceptions about themselves over time or in some cases these views are instilled in them by those around them.

### Self-discrimination

One of the characteristics that the older adultsparticipants express when entering old age was self-discrimination. In fact, these people, in the course of their lives and gaining various experiences, achieve a self-concept that consists of positive and negative dimensions. They believed that they should be a role model for others, and that this passage of life and the difference between this generation and subsequent generations should manifest themselves in a positive way, and in fact they saw themselves as different from others.

*“As you get older, socializing, talking, and studying make you cultivated. If you have an idea about a specific thing, for example, this way of thinking will become more cultivated and will evolve and develop” (M. 16).*

On the other hand, some older adultsparticipants believed that as they enter old age, they suffer from many physical and mental disabilities which can affect their individual and social lives negatively.

*“I cannot travel, but my children insist that I go. I tell them that I cannot join because I’m sick. Because I cannot hold my urine, I cannot go and I say I do not join. I mean I’m not comfortable (participant number 5).*

### Other discriminatory

In addition to the positive and negative discrimination that the older adultsolder adults attribute to themselves, a combination of positive and negative discrimination of those around them is also effective in shaping ageism. In Iranian societies, the older adultsolder adults are highly valued and are considered as chief and seniors of the family or the community. It is in this way that they can experience the feeling of being useful and valuable.

*“I value my grandparents very much, because they are highly experienced and worldly-wise. They are more religiously observant, and as a result they can be a spiritual support” (M. 20).*

On the other hand, a set of negative traits is also attributed to the older adultsolder adults people. Changes in today’s societies threaten the status and value of the older adultsolder adults and have jeopardized the value and dignity of these people as social capital.

*“In family gatherings, most people are the younger members, well, they have different worldviews and hobbies, and these restrictions cause the older person to become withdrawn and want to leave, and does not like to engage in discussions (M. 18). “*

The existence of a discriminatory paradox in the older adultsolder adults with different characteristics causes differences in the experience of ageism in this age group. Therefore, in the next step, an attempt was made to identify the contexts that had arisen following ageism in individuals. The concepts of “family context” and “cultural context” in the present study were relevant in this field.

### Family context

One of the important functions of the family is to play the role of caring for and supporting the older adultsolder adults people. Although the central role of the family in caring for the older adultsolder adults is associated with increasing the health of the older adultsolder adults, but social, economic and technological changes have led to an increased period of old age. This fact has caused changes in values ​​and traditions. Therefore, it has challenged the care of the older adultsolder adults in the family. Thus, family context with two sub-categories of “imposed loneliness” and “emotional support” is another area that is effective in exacerbating or controlling ageism in the older adults.

### Imposed loneliness

With the advent of modernity in Iranian families, there have been changes in the relationships of family members. As the role of children in caring for older adults parents diminishes, their former duties are transferred to complementary organizations, including nursing homes. Iranian society is in a state of transition from traditional to industrial. Despite the fact that people have grown up with traditional ideas, they live in a society that must act economically and socially in accordance with an industrial society. Therefore, despite the support that is given to our older adults, due to the involvement of children with their own work, education and family life, the older adults may be left alone.

Due to the dominant context of Iranian society, living a nursing home requires a lot of infrastructure, and negative reactions to it are often illustrated by the older adults members. In such a way that in most cases, being in these places is not in accordance with the wishes of the older adults and living in it is accompanied by an intensification of feelings of loneliness.

*“Obviously I wanted to be in my own house, my own house is much better. Here (*nursing home*) people get depressed. It is true that they take care of everything right here, but I miss home, I want to go home (M. 23)”*.

### Emotional support

Emotional support has been suggested as an important factor in the mental health of the older adults that can affect other dimensions of a person’s health. Following old age, older people experience less stress if they have enough support from others, especially their children. In fact, the prevailing conditions in Iranian society, which describe the older adults as valuable human beings, create this expectation in these people.

*“My children value me very much. They listen to me, for example, they respect, and they stand up in front me as soon as I enter the room. They kiss me and shake every time they return from work. And I think that this is enough for me.*

On the other hand, ignorance and lack of emotional support is the most important damage that hurts the older adults the most. Because the most important need of the older adults is respect and attention. Just as satisfaction with attention comes with positive consequences, lack of emotional support and love from others, especially children, is associated with negative consequences such as feelings of rejection and emptiness.

*“My grandfather who lives alone, is 84 years old and he is very wealthy and has no financial problems at all, but his children live abroad and his most common complaint is that they do not consider him as a human at all, they do not call him and there is no love (M. 14) “.*

### Cultural context

One of the important issues in how ageism is formed in the older adults people was the role of culture. This role is especially important when it can directly affect other areas such as the ups and downs of old age discussed in the previous section.

#### Values ​​and mindset of society

In traditional Iranian culture, the older adults people are described as valuable members, which is due to their essential role in the transfer of knowledge and experience. This reinforces a sense of self-worth and other-worth. Of course, a realistic approach to current sociological changes in traditional cultures reflects differences in attitudes toward respect for the older adults members of the family.

Nowadays, due to the industrialization of societies, social and economic changes, new lifestyles, technology and difficult living conditions have reduced valuing and respecting the older adults remarkably. Meanwhile, the existence of gender differences that arise from social concepts causes differences in the way society views old men and women. In this way, men are always recognized as a superior power over women.

*“In our family, patriarchy was not very common, but for example, my grandmother herself was someone who was affected by it. I remember when my grandfather passed away, my grandmother was in a state like she really lost her supporter and there I might say there was a form of this patriarchy (M. 19).“*

#### Laws and policies

Today, with the increase in the number of older adults people and the passing of a significant part of people’s lives in the third period of life, the issue of old age has become one of the important issues in the field of policy making. Old age is usually accompanied by numerous changes such as changes in family structures, work patterns, retirement, and changes in health status. The problem begins where, despite being aware of these needs, the social and economic context does not have sufficient capacity to meet the needs of the older adults people as social assets. It is obvious that the lack of proper bedding has made it more difficult for the older adults more than before which leads to the appearance of ageism both at the individual and social levels.

*“The Government’s policies are also 100% effective in discriminating, for example, in the employment debate, people over 40 are not hired, or people over 60 retire early or are not employed. They do not consider their wisdom and life experience but to their aging status and its limitations. (M.25).*

Since some older adults in interviews mentioned that not enough arrangements have been made for them to enjoy this period of life, the existence of environmental and cultural factors is also effective in experiencing healthy and successful aging. They only spend the days of their lives to die. older adultsolder adults*"In some countries, when one reaches a certain age and retires, theyare relieved because they are supported by the society and the government, but in others, when a person reaches 60, 65 years old and then retires, they think there is no other way but to settle and wait for death. It is after this age we feel that the person is useless (M. 25)”.*

### Generation gap

Generation gap is a phenomenon that occurs as a result of social changes. Although this is an obvious fact, its emergence in an Iranian society is evolving and developing into other forms. Thus, continuous change in various aspects of traditional life has revealed values ​​different from traditional norms and beliefs among the new generations. Because these values ​​are not very compatible with the accepted norms of traditional Iranian society. Therefore, it causes a value-normative gap between the present and the past and confronts different generations with different dos and don’ts, thereby widening the gap between them.

*“Especially now there is a difference between most of the older adults and the young. They are from different decades so are we. Maybe my mom didn’t have as much difference in opinions as I have with my grandmother now. During these decades in the meantime, the situation and beliefs have changed a lot. I think it is because of the great speed with which science and technology have changed (M.18)”.*

The transcripts of the interviews and the analysis of the statements based on the participants’ experience indicated that they tried to come up with strategies and activities such as “maintain dynamics”, “socio-cultural care” and “proper health care” at the individual and social level. To overcome this concern. Thus, the sum of these six subcategories under the title of " striving to tackle ageism"” was selected as the most important process in combating ageism in the older adults.

#### ***Maintaining*** Dynamics

being energetic and vitality and efforts to maintain it have influenced various aspects of life, including the psychological, physical and learning aspects of the older adults. In fact, aging is interpreted as maintaining health and being active in the community. This concept also emerged in interviews emphasizing the presence of the older adults in various social settings and focusing on the skills and knowledge they have.

*“I feel that working gives me a state of pride and vitality. When I work, I feel that I am healthy, that is, if, God forbid, I have a disease, I do not feel that I am sick. No matter how hard it is I take a walk and run the home errands myself. (M. 27)”*.

### Socio-cultural care

In order to succeed in tackling discrimination, it is necessary to take individual actions that are in line with social actions. Because the older adults person, as a member of society, still lives in this society, however, a series of events have diminished their social role compared to previous periods of their life. Therefore, paying attention to the power and strength of the older adults and trying to preserve them in society and assigning appropriate responsibilities to their age can be effective in honoring them. On the other hand, establishing a connection between the two generations will provide an opportunity to transfer the experiences of the older adults to younger age groups,. There is a need to create a culture and grounds for delegating responsibility to the older adults to do things to the best of their ability. 1According to the participants of the present study, involving them in social spheres and delegating responsibilities with regard to their physical condition will increase their sense of worth and usefulness.

*“The older adults should be held accountable, both for their personal affairs and for the serious decisions about the older adults themselves. For example, the things that can be done by themselves, even if it takes time to do so” (M. 20).*

### Proper medical care

One of the points that the older adults themselves mentioned was the provision of different health services offered to them by the health care system; This means that in the health care system, young people, unlike the older adults, who are often considered as a burden on the society and only add to the government’s medical expenses, have a higher priority in receiving health care and services because they considered as useful and efficient assets of society. Therefore, it is necessary to take measures in this regard so that the older adults, like other age groups, benefit from medical services and experience healthy aging.

**Fig. 1:**
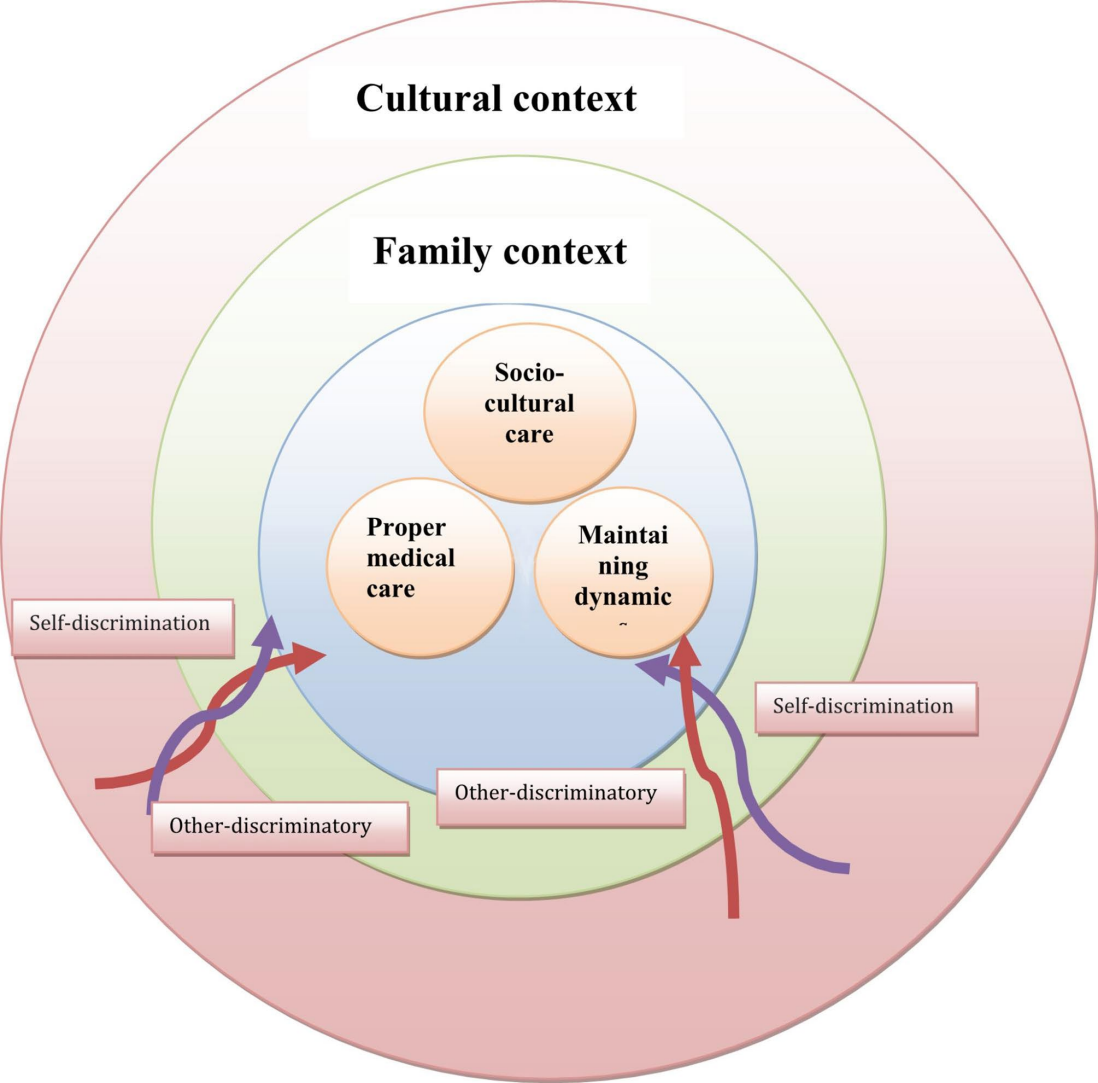
Theoretical model of the process of ageism in the elderly people

“Especially in the context of the corona pandemic, we can easily notice that the older adults are among the groups with a high incidence. This culture should be created, and fortunately, contrary to popular belief, the older adults are also benefiting from these services. Even in the form of specialized treatments or the allocation of beds to them. Even the fact that the covid-19 vaccine was distributed to several medical centers and given priority to the older adults can have good signs of attention to the older adults” (M. 22). Figure 1 shows Theoretical model of the process of ageism in the older adults people.

## Discussion

The results of data analysis demonstrated that the process of ageism is contextual and also varies according to the individual characteristics of the older adults. The conditions created after old age were identified with concepts such as family context and cultural context. The interaction of these concepts with each other all indicated the need of the older adults to pay attention to different physical, mental and psychological dimensions. For this reason, fear of loneliness and rejection was identified as the most important concern of the older adults. Although its severity varied among different older adults with different characteristics (physically, mentally, and economically), all of them agreed on it. The older adults also did their best to overcome the discrimination that they rightly or wrongly understood and tolerated and were imposed on them.

Based on the results of the present study, it was found that ageism in the Iranian older adults is a complex field that includes cognitive, behavioral and emotional effects. In addition, this concept highlights social inequalities because it is more focused on older women and the poor [25]. Because of the loss of physical and mental power in the elderly creates various challenging issues that necessitate the need for planning in order to provide services to this population [3]. In this regard, although positive changes have occurred, the elderly remain somewhat vulnerable [3]. The results of a qualitative study in this field also demonstrated that some retired older adults people as a result of ageism prevent themselves from taking advantage of opportunities for promotion, education and employment in other professions [26]. The situation in Iranian society is such that people on the verge of retirement do not receive the necessary psycho-social training and may be left alone after retirement.

The family context and the environment in which the older adults are present and spend the rest of their lives are important factors that are effective in creating a sense of security, respect, insecurity and other psychological consequences. In the present study, based on the interviews, the family context includes subcategories such as “imposed loneliness” and “emotional support”. Loneliness becomes a serious and important issue at some point in life, i.e. old age [27]. One study found that old people who experienced loneliness were neglected and abandoned [28]. Regarding the results of the present study, the point made about the subcategory of imposed loneliness from the perspective of most study participants was that loneliness was taken for granted. In fact, there was a belief among the participants that loneliness was considered as something normal that should be accepted and one must get along with it. The results of a longitudinal study also indicated that there is an expectation among the older adults that loneliness is part of the aging process and by emphasizing ageism which considers the older adults as people in need of help and forgotten, it was concluded that measures should be taken to reduce this feeling in these people [29].

On the other hand, increasing the number of the older adults calls attention on the need to pay more attention to emotional and social support as one of the effective factors in improving the mental and physical health of the older adults and it also highlights paving the way for a successful aging experience [30]. However, there are still traditional expectations and approaches to receiving parental support from parents [31]. What the researchers found in this study regarding emotional support was that the cultural factor plays a significant role in receiving this type of support. In Western cultures, the older adults are more likely to provide services and support, while in Eastern societies, because of the educational teachings in these cultures, children should respect their parents and elders. They have a heavy responsibility towards them in this time; they tend to receive support from their children. Also, the results of other studies indicated that limited emotional support has more serious negative effects on the mental health of older adults people in Asian countries than in Western societies because of interdependency. Family solidarity and empathy are very valuable [32]. Nazari et al. (2018) in their study on social support in the Iranian older adults reported that the older adults, even though they tend to receive support from children and friends, this expectation is higher from children [33]. Therefore, receiving support for the older adults is of particular importance, because old age is associated with an increase in exposure to various stressors, including the incidence of chronic diseases and functional limitations, loss of income sources and the loss of a spouse [31].

In the present study, in the face of the phenomenon of aging and ageism, culture is considered as an element that is influenced by values ​​and ways of thinking, laws and policies and generation gap that are effective in forming ageism. One of the important prevalent issues of today’s societies, despite globalization and cultural changes, is the discussion of gender stereotypes [34]. In the present study, older adults women with lower economic and social status also referred to the fact that “man was the little god of woman” and would lose a large part of their credibility by losing their husbands. And are more discriminated against. As long as social stereotypes are present in any culture, depending on their power, they are an obstacle to women achieving appropriate social goals and status [35]. In developing countries like Iran it is considered a value, but in the next few decades, due to demographic, cultural, social and economic changes, governments must seek to formulate policies and programs to develop formal care, because we are currently facing an institutional care crisis. As the number of older adults people in need of formal care is increasing, so are accommodation facilities for the older adults, and this will become more challenging in the coming decades [36].

Negative attitudes toward the older adults lead to discrimination in care and the emergence of stereotyped and negative behaviors by caregivers. In the present study, not only some older adults people acknowledged this behavior of the health care system, but also policy-makers and experts in this field mentioned this refusal and discrimination of care as a matter of course and ageism. In other words, despite the severe needs of the older adults for care, the lack of quantitative and qualitative care leads to deteriorating health and even death in this age group [37]. Since old times there has usually been disagreement and conflict between the two generations, but this disagreement has never been a serious issue until the emergence of the new industrial society. This difference has become more apparent since the twentieth century due to the drastic changes in human societies. The reason for such a gap is the existence of defects or disorders in the process of socialization [38].

According to the present study, the older adults were trying to bring their behaviors closer to the performance of young people so that they could have better and stronger relationships with them and be less discriminated against. Research on motivational development has shown that young people tend to seek greater achievement, while older people are more motivated to maintain performance and ability and prevent negligence and being forgotten and also experiencing loss [39]. In the present study, one of the strategies used by the older adults for their dynamism and efforts to eliminate discrimination was to try to play their current roles or even to accept new ones. According to Van Dyk (2014), recent demographic changes in terms of population aging are a threat to society and economic structures - in which it is imagined “being reproductive and staying active” equals “staying young” as an “acceptable entity.“ [40].

As the older adults population grows, the number of people in this age group who will be hospitalized will increase. However, according to various studies, the desire to care for young patients is one of the issues in providing medical services that leads to discrimination in the care of the older adults [41]. The findings of the study by Hosseini et al. (2020) indicated that the evaluation of care for the older adults by family members, society and the care provider system has a positive effect on the acceptance and provision of quality care for the older adults [42]. The results of another study in this field indicated that effective supervision of geriatric specialist in the training of experienced personnel, spending enough time and money can be effective in providing quality care and prevent the marginalization of this age group in the health care system [10]. Liu also stated in his findings that having a positive attitude towards the older adults has led to the creation of a management system in the hospital that reduces ageism [43].

Therefore, the study of texts indicated that although ageism is a matter of cultural structure and context but there are commonalities in the causes and factors that cause and aggravate it. In the present study, the most important concerns of the older adults were identified as “fear of loneliness and rejection”.

## Conclusion

The process of ageism in the older adults is context-dependent and varies according to each individual’s condition, causing older people to experience varying degrees of it. Fear of loneliness and rejection is influenced by factors such as family context and cultural context, which were identified as the most important concerns and worries of the older adults. The older adults used a set of strategies to deal with this fear of being alone. Efforts to eliminate discrimination, which included measures such as “maintain dynamics”, “socio-cultural care” and “proper health care”, were identified as the most important processes for the older adults to deal with ageism. The attempt to tackle ageism against the older adults was meant to preserve their existential and personal identities.

## Data Availability

The data are not publicly available due to privacy or ethical restrictions. The data that support the findings of this study are available from the corresponding author upon reasonable request.

## Footnotes

1 .

## References

[1] Kazemi A (2021). Caregiver burden and coping strategies in caregivers of older patients with stroke. BMC Psychol.

[2] Mejía ST (2017). Successful aging as the intersection of individual resources, age, environment, and experiences of well-being in daily activities. Journals of Gerontology Series B: Psychological Sciences and Social Sciences.

[3] Rashedi V et al. *Ageism among primary health care professionals and nurses in Iran* Ethics, Medicine and Public Health, 2021. 17: p. 100638.

[4] Polat Ü (2014). Nurses’ and physicians’ perceptions of older people and attitudes towards older people: Ageism in a hospital in Turkey. Contemp Nurse.

[5] Kang H, Kim H (2022). Ageism and psychological well-being among older adults: a systematic review. Gerontol Geriatric Med.

[6] Hu RX (2021). Associations of ageism and health: a systematic review of quantitative observational studies. Res aging.

[7] ŞAHİN FT, BAYRAKTAR E, ERTEN ZK. Determination of the Attitudes of the Health Staff Working for the institutions providing primary Health Care towards Ageism. Volume 8. Hacettepe Üniversitesi Hemşirelik Fakültesi Dergisi; 2021. pp. 167–77. 2.

[8] Cary LA, Chasteen AL, Remedios J (2017). The ambivalent ageism scale: developing and validating a scale to measure benevolent and hostile ageism. Gerontologist.

[9] Gholamzadeh S (2022). Age discrimination perceived by hospitalized older adult patients in Iran: a qualitative study. Health Promotion Perspectives.

[10] Mehri S et al. *Explaining Nurses’ Perception of the Causes of Ageism in Hospital Settings*.Electronic Journal of General Medicine, 2020. 17(5).

[11] Thomas R. *Ageism: The perceptions and experiences of the elderly in Kerala*. 2016.

[12] Rahmaniah BI, Krisnatuti D (2016). The perception of Ageism, Generativity, and the attainment of Developmental Tasks of Elderly Widowers and Widows in Bogor, West Java, Indonesia. J Family Sci.

[13] Ayalon L (2018). Perceived age discrimination: a precipitator or a consequence of depressive symptoms?. The Journals of Gerontology: Series B.

[14] Bai X, Lai DW, Guo A (2016). Ageism and depression: perceptions of older people as a burden in China. J Soc Issues.

[15] Zhang X (2020). Negative self-perception of aging and mortality in very old chinese adults: the mediation role of healthy lifestyle. The Journals of Gerontology: Series B.

[16] Ng R, Chow TYJ, Yang W (2022). The impact of aging policy on societal age stereotypes and ageism. Gerontologist.

[17] Vickerstaff S, van der Horst M (2022). Embodied ageism:“I don’t know if you do get to an age where you’re too old to learn. J Aging Stud.

[18] Band-Winterstein T (2015). Health care provision for older persons: the interplay between ageism and elder neglect. J Appl Gerontol.

[19] Podhorecka M (2022). Attitudes towards the Elderly in Polish Society: is knowledge about Old Age and Personal Experiences a predictor of Ageism?. Psychol Res Behav Manage.

[20] Phelan A (2018). The role of the nurse in detecting elder abuse and neglect: current perspectives. Nursing: Res Reviews.

[21] Ng R, Indran N, Liu L. Ageism on Twitter during the COVID-19 pandemic. Journal of Social Issues; 2022. 10.1111/josi.12535 PMC9349453 35942488

[22] Navab E, Negarandeh R, Peyrovi H (2012). Lived experiences of iranian family member caregivers of persons with Alzheimer’s disease: caring as ‘captured in the whirlpool of time’. J Clin Nurs.

[23] Rababa M, Al-Sabbah S, Bani-Hamad D (2022). Nurses’ death anxiety and ageism towards older adults amid the COVID-19 pandemic: the moderating role of symbolic immortality. Geriatrics.

[24] Singh S, Estefan A (2018). Selecting a grounded theory approach for nursing research. Global qualitative nursing research.

[25] Ayalon L, Tesch-Römer C. Taking a closer look at ageism: self-and other-directed ageist attitudes and discrimination. Springer; 2017. pp. 1–4. 10.1007/s10433-016-0409-9 PMC5550624 28804389

[26] Van der Horst M (2019). Internalised ageism and self-exclusion: does feeling old and health pessimism make individuals want to retire early?. Social Inclusion.

[27] Ramamonjiarivelo Z, et al. *Assessing the effectiveness of intergenerational virtual service-learning intervention on loneliness and ageism: a Pre-Post Study*. In *Healthcare*. MDPI; 2022. 10.3390/healthcare10050893 PMC9141066 35628031

[28] HERAVI KM et al. *Loneliness from the perspectives of elderly people: A phenomenological study* 2008.

[29] Gibney S, Moore T, Shannon S. Loneliness in later life: a cross-sectional survey analysis of place-based factors in Ireland. Quality in Ageing and Older Adults; 2019.

[30] Cha K-S (2019). The level of successful aging and influencing factors of the community elderly. Korean J Health Promotion.

[31] Tajvar M, Grundy E, Fletcher A (2018). Social support and mental health status of older people: a population-based study in Iran-Tehran. Aging Ment Health.

[32] Lim LL, Kua E-H. *Living alone, loneliness, and psychological well-being of older persons in Singapore* Current gerontology and geriatrics research, 2011. 2011. 10.1155/2011/673181 PMC3182578 21969827

[33] Nazari S (2016). Perceived social support in iranian older adults: a qualitative study. Educ Gerontol.

[34] Hatch LR (2005). Gender and ageism. Generations.

[35] Montepare JM, Zebrowitz LA. *A social-developmental view of ageism* 2002.

[36] Shirali E, Shahbazi M (2019). Presenting Welfare Services to Elderly in selected Country and Lessons for Iran. Social Secur J.

[37] Mehri S (2020). Clarification of ageism in the care system. Iran J Ageing.

[38] Blanche-T D, Fernández-Ardèvol M (2022). (Non-) politicized ageism: exploring the multiple identities of older activists. Societies.

[39] Nikitin J, Schoch S, Freund AM. *The role of age and motivation for the experience of social acceptance and rejection* Developmental Psychology, 2014. 50(7): p. 1943. 10.1037/a0036979 24842461

[40] Van Dyk S (2014). The appraisal of difference: critical gerontology and the active-ageing-paradigm. J Aging Stud.

[41] Brown LG, Wang CH (2022). Dismantling ageism among nursing students. Teach Learn Nurs.

[42] Hosseini MA (2020). Combating with ageism in care settings: a qualitative study. EurAsian J Biosci.

[43] Lui NL, Wong CH (2009). Junior doctors’ attitudes towards older adults and its correlates in a tertiary-care public hospital. Annals Acad Med Singap.

